# Transcriptional comparison of adult human primary Retinal Pigment Epithelium, human pluripotent stem cell-derived Retinal Pigment Epithelium, and ARPE19 cells

**DOI:** 10.3389/fcell.2022.910040

**Published:** 2022-08-26

**Authors:** Elke K. Markert, Holger Klein, Coralie Viollet, Werner Rust, Benjamin Strobel, Stefan G. Kauschke, Bar Makovoz, Heike Neubauer, Remko A. Bakker, Timothy A. Blenkinsop

**Affiliations:** ^1^ Global Computational Biology and Digital Sciences, Boehringer Ingelheim Pharma GmbH, Biberach, Germany; ^2^ Translational Medicine and Clinical Pharmacology, Boehringer Ingelheim Pharma GmbH, Biberach, Germany; ^3^ CardioMetabolic Diseases Research, Boehringer Ingelheim Pharma GmbH, Biberach, Germany; ^4^ Ophthalmology Cell Development and Regenerative Biology, Black Family Stem Cell Institute, Icahn School of Medicine at Mount Sinai, New York, NY, United States

**Keywords:** retinal pigment epithelium, iPSC-RPE, ARPE-19, epithelial to mesenchymal transition, age-related macular degeneration, proliferative vitreoretinopathy, RNA-sequencing, transplantation

## Abstract

The therapeutic potential of pluripotent stem cells is great as they promise to usher in a new era of medicine where cells or organs may be prescribed to replace dysfunctional tissue. At the forefront are efforts in the eye to develop this technology as it lends itself to *in vivo* monitoring and sophisticated non-invasive imaging modalities. In the retina, retinal pigment epithelium (RPE) is the most promising replacement cell as it has a single layer, is relatively simple to transplant, and is associated with several eye diseases. However, after transplantation, the cells may transform and cause complications. This transformation may be partially due to incomplete maturation. With the goal of learning how to mature RPE, we compared induced pluripotent stem cell-derived RPE (iPSC-RPE) cells with adult human primary RPE (ahRPE) cells and the immortalized human ARPE-19 line. We cultured ARPE-19, iPSC-RPE, and ahRPE cells for one month, and evaluated morphology, RPE marker staining, and transepithelial electrical resistance (TEER) as quality control indicators. We then isolated RNA for bulk RNA-sequencing and DNA for genotyping. We genotyped ahRPE lines for the top age-related macular degeneration (AMD) and proliferative vitreoretinopathy (PVR) risk allele polymorphisms. Transcriptome data verified that both adult and iPSC-RPE exhibit similar RPE gene expression signatures, significantly higher than ARPE-19. In addition, in iPSC-RPE, genes relating to stem cell maintenance, retina development, and muscle contraction were significantly upregulated compared to ahRPE. We compared ahRPE to iPSC-RPE in a model of epithelial-mesenchymal transition (EMT) and observed an increased sensitivity of iPSC-RPE to producing contractile aggregates *in vitro* which resembles incident reports upon transplantation. P38 inhibition was capable of inhibiting iPSC-RPE–derived aggregates. In summary, we find that the transcriptomic signature of iPSC-RPE conveys an immature RPE state which may be ameliorated by targeting “immature” gene regulatory networks.

## Introduction

Age-related macular degeneration (AMD) impairs vision by the loss of retinal pigment epithelium (RPE) and photoreceptors in the region of the macula. AMD is the leading cause of blindness, affecting over 200 million individuals worldwide (Wong et al., 2014). RPE dysfunction precedes degeneration of the neural retina and vision loss due to many supportive roles that RPE play for photoreceptors and phototransduction (M'Barek et al., 2019). Some of the roles include the formation of the blood–brain barrier, absorption of stray light, supply of nutrients, and recycling of visual pigment, (Strauss, 2005). Consequently, the loss of RPE eventually leads to the loss of photoreceptors and irreversible blindness. AMD can be divided into two groups defined by the presence of choroidal neovascularization (wet AMD) or its absence (dry AMD) (Bird et al., 1995). Palliative treatment options are available for the “wet” form of the disease with choroidal neovascularization, including antineovascular agents, photodynamic therapy, and thermal laser therapy (Ammar et al., 2020). The gold standard of treatment for wet AMD is the use of anti-VEGF antibodies. However, there are no current treatments for the more widespread, dry AMD aside from the recommendation of oral supplementation of antioxidants (Age-Related Eye Disease Study Research, 1999). Wet AMD patients have much more severe photoreceptor degeneration, while patients with dry AMD still maintain photoreceptors and therefore are a promising patient subgroup to benefit from RPE cell replacement therapy (Gehrs et al., 2006).

Cell therapy can potentially stop or reverse AMD by replacing degenerated RPE, thereby restoring retinal function and vision. Autologous RPE/choroid transplant attempts from the peripheral to central retina have demonstrated partial restoration of vision in AMD patients (Stanga et al., 2002). However, autologous transplantation is limited by the complexity of surgery and the lack of technique adoption (Cereda et al., 2010; Parolini et al., 2020). Pluripotent stem cells have been proposed to be an attractive alternative cell source for transplantation (Shi et al., 2017). Pluripotent stem cells can indefinitely self-renew and differentiate into any cell type found in the adult body, making them a promising source to generate unlimited RPE for cell therapy (Takahashi and Yamanaka, 2006).

Recent human clinical trials and some preclinical trials have noted a tendency to produce epiretinal membranes upon pluripotent stem cell-derived RPE transplantation. One group transplanted human embryonic stem cell–derived RPE in two patients and showed visual improvement. However, they also reported the production of epiretinal membranes (da Cruz et al., 2018). Another group used HLA-matched allogeneic-induced pluripotent stem cell–derived RPE transplanted into patients, and reported epiretinal membrane production as adverse events in two out of five individuals (Sugita et al., 2020). Finally, in a Phase I/IIa clinical trial using human embryonic–derived RPE, epiretinal membranes were noted in a subset of patients (Banin et al., 2017).

This common adverse event may originate from various sources. The epiretinal membrane formation may originate from the surgical approaches used for cell; they may be due to the known tendency for RPE to produce epiretinal membranes as in the case with proliferative vitreoretinopathy, or they may be due to an epigenetic plasticity resulting from incomplete differentiation in vitro form the pluripotent stem cells. There are many studies that have carefully examined RPE physiology from pluripotent sources demonstrating that they do exhibit RPE function and therefore are bona fide RPE (Liao et al., 2010; Ferrer et al., 2014). There has also been some evidence of plasticity after the differentiation of iPSC into RPE, particularly after three passages (Singh et al., 2013). We sought to compare the gene expression profiles between a collection of ahRPE, the well-known immortalized RPE cell line ARPE-19, and two iPSC-RPE lines with the intent to uncover what differences may persist between pluripotent stem cell–derived RPE and ahRPE (Salero, 2012; Schiff L., 2019).

## Results

**FIGURE 1:**
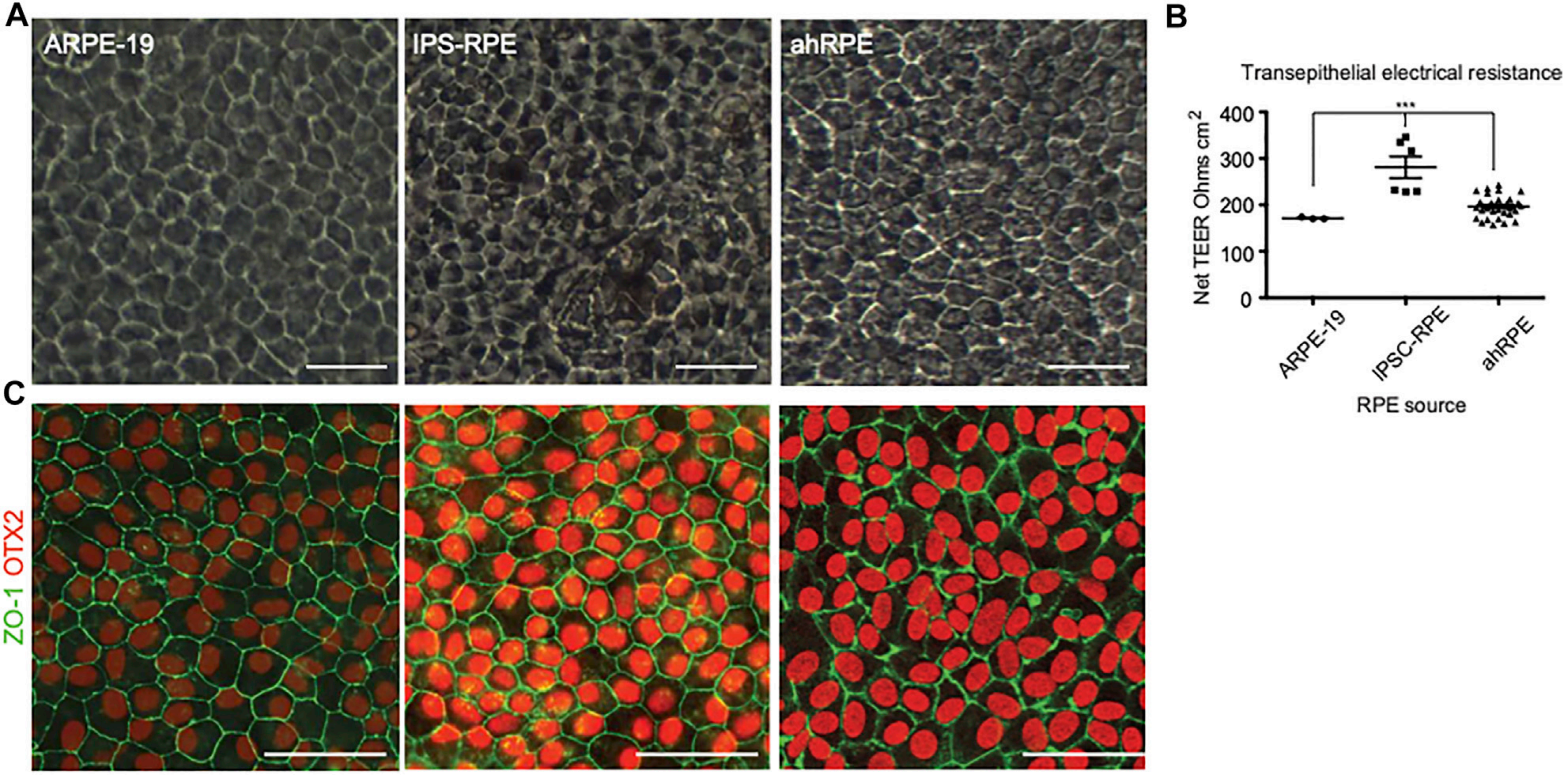
Phenotypic comparison between different sources of RPE. **(A)** Phase images of RPE after 1 month in culture. **(B)** Sentinel cultures of RPE plated in A were also plated in transwells, and TEER was measured after 1 month in culture. **(C)** Immunofluorescence of RPE from the different sources stained with antibodies to RPE markers OTX2 and ZO-1. *** indicates *p*-value is > 0.0001. Scale bar = 50 μm.

ARPE-19, iPSC-RPE, and ahRPE were cultured for one month. Phase images were taken from all samples, and transepithelial electrical resistance (TEER) was measured at the 1-month time point. Cobblestone morphology was scored by the eye from 1 to 5, with 5 representing a regular hexagonal and even cobblestone morphology and 1 representing elongated fibroblasts. Some samples were fixed and stained for the confirmation of expression of RPE markers. The rest of the samples were processed to extract RNA, and whole transcriptome RNA-sequencing was performed in a total of three ARPE-19 samples (replicates), six iPSC-RPE samples from two donors (three differentiation rounds per donor), and 23 ahRPE samples from 23 human donors. All human donor cells used were at population doubling six to seven, which correlates to the expansion and analysis demonstrating native physiology (Blenkinsop et al., 2015). Cell lines were also genotyped for polymorphisms associated with elevated risk of AMD and PVR (Table 1). Representative examples of the cultures from ARPE-19, iPSC-RPE, and ahRPE exhibiting a regular RPE cobblestone morphology are presented in Figure 1A. After 1 month, the net TEER was measured as a quality check to confirm barrier function (Figure 1B). ARPE-19 exhibited a mean TEER of 171.3 ± 1.33 SEM, *n* = 3, iPSC-RPE showed a mean TEER of 281.0 ± 23.3 SEM, *n* = 6, and for ahRPE, we recorded a mean TEER of 196.4 ± 4.12 SEM, *n* = 23. Each group was significantly different from each other, with a *p*-value <0.0001. An additional validation of RPE identity was conducted by immunostaining with the RPE markers of identity and maturity OTX2 and ZO-1 (Figure 1C).

After 1 month in culture, ARPE-19, IPSC-RPE, and ahRPE were processed for bulk RNA-seq and genotyping. RNA was isolated and purified using the Qiagen AllPrep DNA/RNA kit. The samples were then tested for RNA quality using the Agilent bioanalyzer. All samples passed initial RNA quality control with RNA Integrity Numbers (RIN) between 8.5 and 9.8 and were accepted for sequencing (see Methods for details). Data were processed from raw reads using a modified version of the ENCODE “Long RNA-Seq” pipeline, followed by custom analyses in R (see Methods for details). We analyzed the variability in the dataset by principal component analysis (PCA).

**FIGURE 2:**
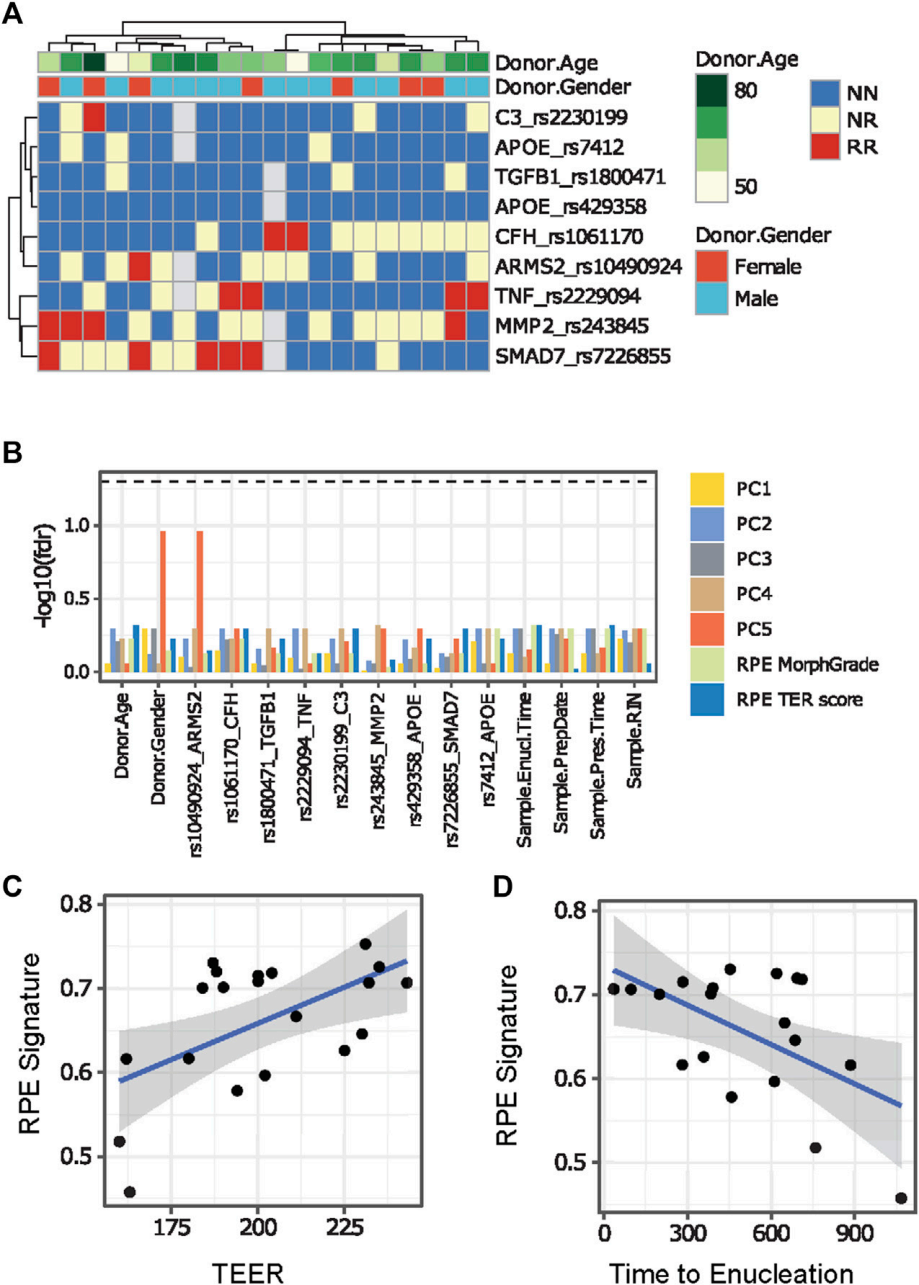
Comparison between ahRPE characteristics and RPE physiology. **(A)** AhRPE were genotyped for high-risk alleles associated with AMD and PVR. **(B)** Potential technical confounders, donor characteristics, and genomic status of ahRPE samples were compared to RNA-sequencing principal component values and physiological sample traits. **(C)** AhRPE TEER measurements were graphed against the RPE signature score of each sample. **(D)** AhRPE sample time of death to enucleation were graphed against their RPE signature score. PC = principal component, NN = homozygous nonrisk, RR = homozygous risk, NR = heterozygous.

We evaluated donor age, sex, time from death to enucleation and time from death to preservation in the obtained ahRPE samples and removed 3 ahRPE samples from further analysis due to sex mismatch. We then examined the remaining set of 20 ahRPE to determine whether donor characteristics or experimental conditions predicted RPE quality. We genotyped the RPE for the presence of SNPs which confer an increased risk to AMD and PVR (Figure 2A). We tested the differential expression associated with each of the genotyped SNPs by building linear models using the limma package. We also tested associations between the genotype and gene expression in an eQTL analysis. Due to the genomic variability among the samples (Figure 2B), these analyses were underpowered and did not yield conclusive results. We did not find a significant correlation between the factors evaluated and principal components using an ANOVA test (Figure 2B). We also did not find a significant relationship between the genotype and RPE morphology or TEER measurements (Figure 2B). Previous bulk RNA-seq analysis of fetal and ahRPE identified 154 genes with a greater than 10-fold elevation of expression than other tissues (Strunnikova et al., 2010). We used this gene set to evaluate whether we can identify trends in any of the measurements taken, which included morphology grade, TEER, and donor information such as enucleation time, preparation time, donor age, donor sex, and genotype. We calculated single sample gene set variation analysis (GSVA) scores for the RPE signature for each sample and found that the RPE signature positively correlated with increased TEER (Figure 2C). We also found that the RPE signature negatively correlates with time between death to enucleation (Figure 2D).

**FIGURE 3:**
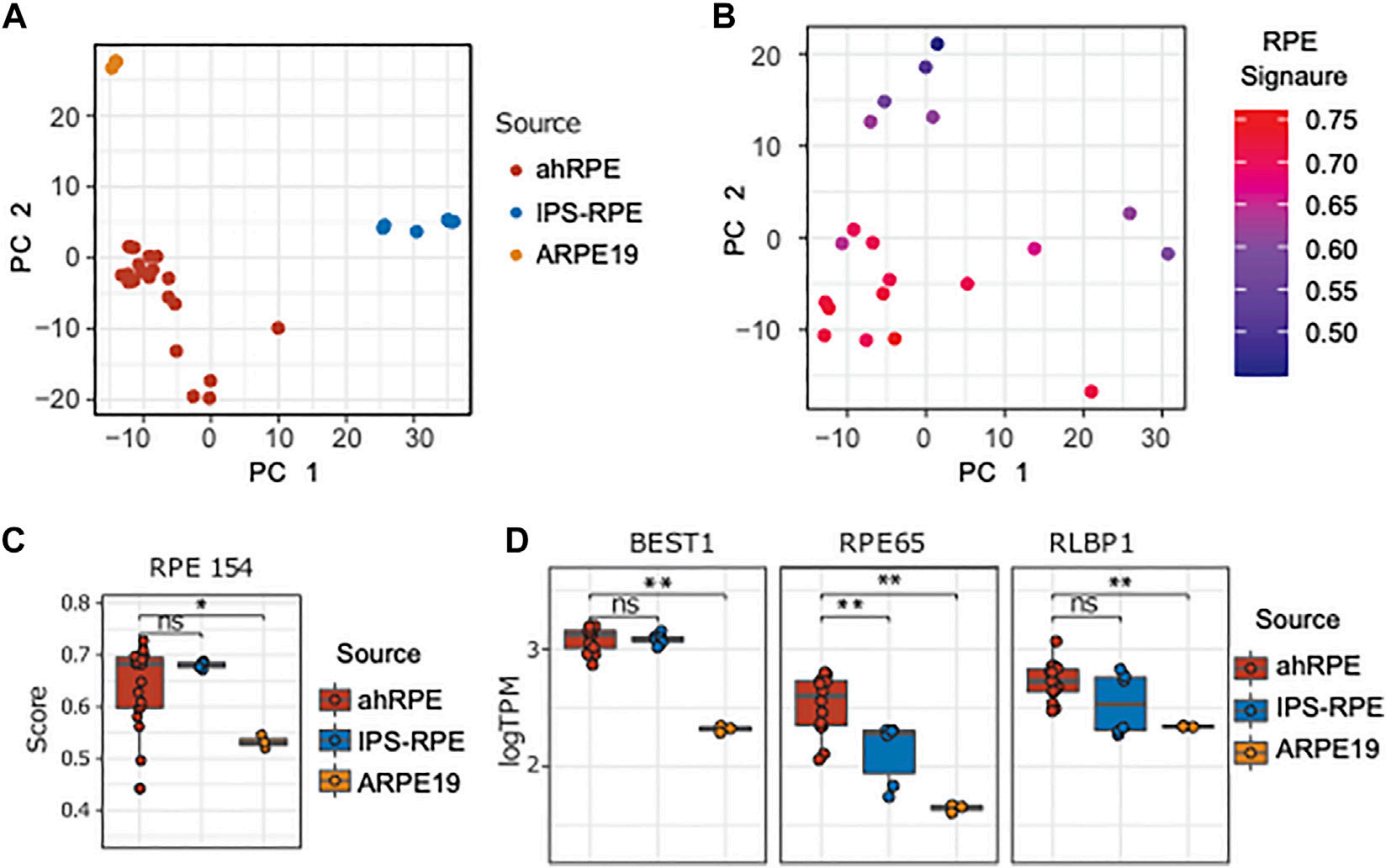
Comparison of ahRPE, ARPE-19, and iPSC-RPE transcriptomes. **(A)** Principal component analysis of RPE samples shows clean separation of source types. **(B)** Principal component analysis for ahRPE samples, with color indicating RPE signature expression for each sample (ssGSEA). **(C)** RPE signature score comparison between ahRPE, iPSC-RPE, and ARPE-19. **(D)** Relative expression of selected genes with RPE-specific functions from different RPE sources. ** = *p*-value < 0.01. PC = principal component.

Next, we performed a principal component analysis of the ahRPE, ARPE-19, and iPSC-RPE dataset (Figure 3A). ARPE-19 samples cluster very tightly together, followed by a tight grouping of the iPSC-RPE. ahRPE exhibit the most expression variation, consistent with the expected human heterogeneity. Despite human genetic diversity, roughly 80% of these samples are tightly grouped, while the remaining samples are distributed at larger distances. To understand this variation, we performed the PCA on the ahRPE samples alone. Notably, the RPE signature scores correlated well with the principal components, indicating that much of the variation in the ahRPE expression data captures RPE tissue–specific expression and molecular RPE quality (Figure 3B). We then compared the RPE signature scores in ahRPE with those of iPSC-RPE and ARPE-19 and found that iPSC-RPE express the RPE signature at a similar level as ahRPE, while ARPE-19 has significantly lower overall expression. This observation is consistent with a lower TEER measured in ARPE-19 transwells (Figure 3C). Some examples of signature genes commonly used as RPE markers and their respective expression from the RPE sources are plotted in Figure 3D. The 154 RPE gene signature expression scores can therefore stand in as a molecular measure of RPE tissue identity and quality.

**FIGURE 4:**
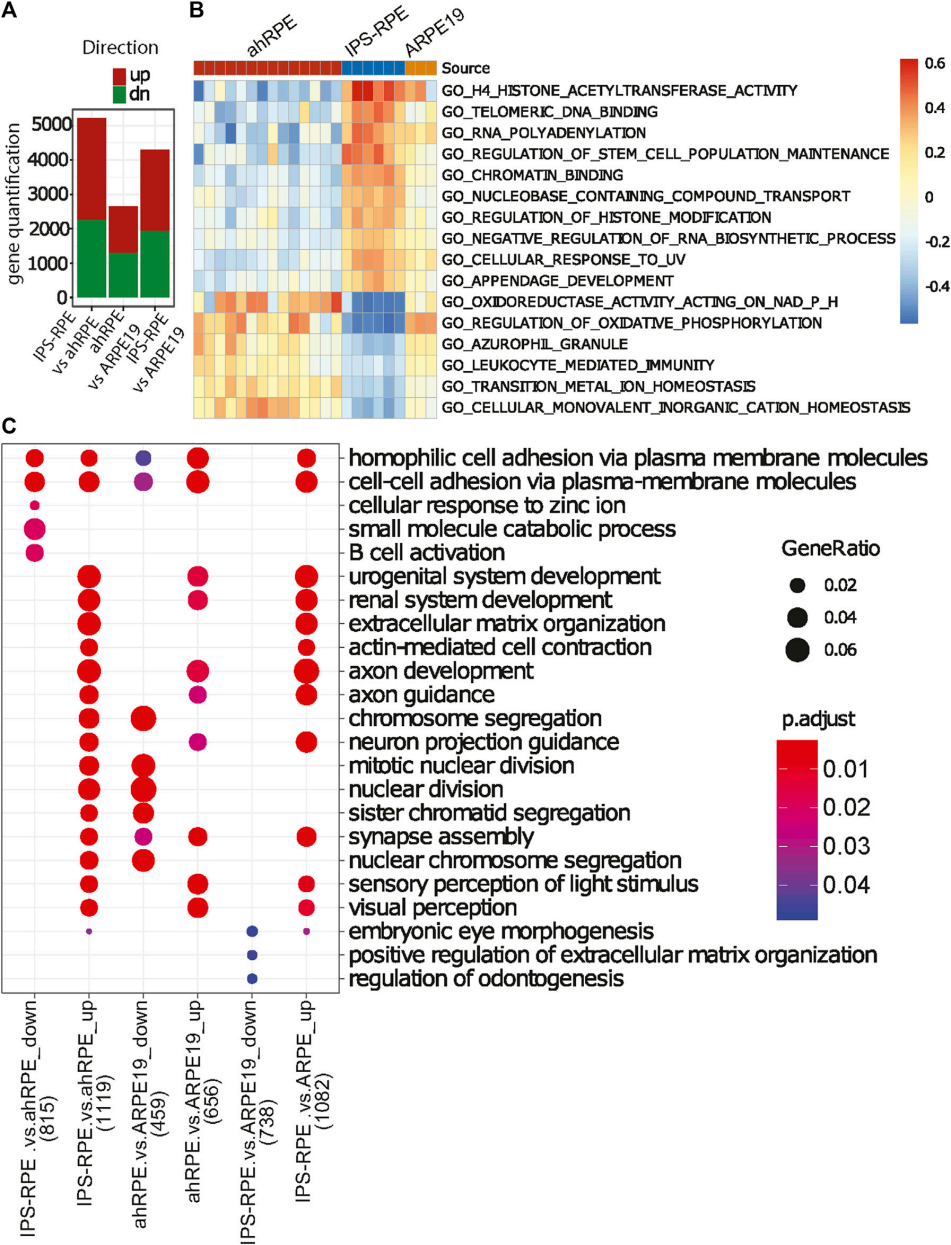
Functional analysis of gene expression differences between the different RPE sources. **(A)** Direct comparison of quantification of total genes upregulated or downregulated with respect to each RPE source (adjusted *p*-value<0.05, |logFC|>0.5). **(B)** Heatmap of relative gene expression in functional categories significantly different between ahRPE and iPSC-RPE. ARPE-19 data included for comparison. **(C)** GO term enrichment analysis of differential gene expression sets (*p* < 0.05) for the comparisons of RPE sources. Top five most significant enrichment terms in for each gene list are included. Number of genes included in analysis in *x*-axis labeled in parentheses.

We focused our analysis on the differences of ahRPE and iPSC-RPE in order to better understand the differences captured in the PCA (Figure 3A). Due to the variability in human RPE quality, we established quality thresholds for the ahRPE samples to ensure inclusion of only bona fide RPE. We excluded a total of six samples with a TEER score less than 175 (equivalent to morphology grade less than 4), donor age above 75 years, or RPE signature score below 0.6. Using the remaining 14 quality ahRPE samples, we performed differential gene expression analysis and evaluated the numbers of differentially expressed genes in the different RPE sources (Figure 4A). We found that many more genes were upregulated in iPSC-RPE relative to ahRPE than the reverse. ahRPE and ARPE-19 possessed roughly similar numbers of upregulated genes. Again, iPSC-RPE possessed a larger number of upregulated genes when compared to ARPE-19. Together, this suggests that iPSC-RPE express more transcripts in general. Indeed, at several thresholds, iPSC-RPE expressed significantly more transcripts in total than ahRPE and ARPE-19 (Supplementary Figure 1A). This effect is not due to differences in RIN (Supplementary Figure 1B). An overlap analysis also showed highly significant overlaps between the differentially expressed gene lists. A significant percentage of the genes that are higher in iPSC-RPE compared to ahRPE are also higher in iPSC-RPE compared to ARPE-19 (Supplementary Figure 2), consistent with the idea that iPSC-RPE express more transcripts than the other two sources. To understand the function of the additional expressed genes in iPSC-RPE, we performed gene ontology comparison of the genes significantly overexpressed in iPSC-RPE vs. ahRPE, in two different approaches. We first used single sample gene set variation analysis (GSVA) to estimate the ranked expression of all gene ontology (GO) terms in each sample. GSVA scores for each term were then tested for significant differences between the groups. Significantly different terms were filtered by fold change and adjusted *p*-values, and the top 15 results were plotted in a heatmap (Figure 4B). The top GO terms overexpressed in iPSC-RPE compared to those of ahRPE are primarily associated with epigenetic regulation including histone acetyltransferase activity, regulation of histone modification, regulation of stem cell maintenance, negative regulation of RNA biosynthetic process, and appendage development. The functions identified as enriched with ahRPE relative to iPSC-RPE included oxidoreductase activity, regulation of oxidative phosphorylation, azurophil granule, transition metal ion homeostasis, and cellular monovalent inorganic cation homeostasis. Therefore, the higher number of expressed transcripts observed in iPSC-RPE samples may indeed be explained by an increased activity of epigenetic regulation and more open chromatin. Conversely, the pathways with elevated expression in ahRPE compared to iPSC-RPE are associated with functions relating to mitochondria and lysosomes. These pathways may be the key to nudging iPSC-RPE toward more closely resembling mature RPE. To explore the differences between RPE cell sources further we used the gene lists derived from the differential expression (DE) analysis between all the RPE sources and analyzed their enrichment in the GO biological process (BP) categories using clusterProfiler (Figure 4C). In particular, up- and downregulated lists were created for each comparison under a fold-change cutoff (|logFC|>1). While the GSVA analysis takes all genes into account (ranking based approach), this analysis ignores all but the selected DE genes in each comparison. Using this approach, the genes that were downregulated in iPSC-RPE, compared to ahRPE, were associated with cell–cell contact, ion regulation, B-cell activation, and catabolism. Genes upregulated in iPSC-RPE compared to ahRPE were associated with renal function, muscle contraction, axon guidance, embryonic eye development, mitochondria nuclear division, and nuclear chromosome segregation. Overall, both analyses suggest similar differences between iPSC-RPE and ahRPE. While RPE signature genes are expressed similarly in both iPSC and ahRPE, iPSC-RPE also express genes involved in early eye development, proliferation, and open chromatin. Gene transcripts enriched in ahRPE function in cell–cell contact maintenance and metabolic processes.

**FIGURE 5:**
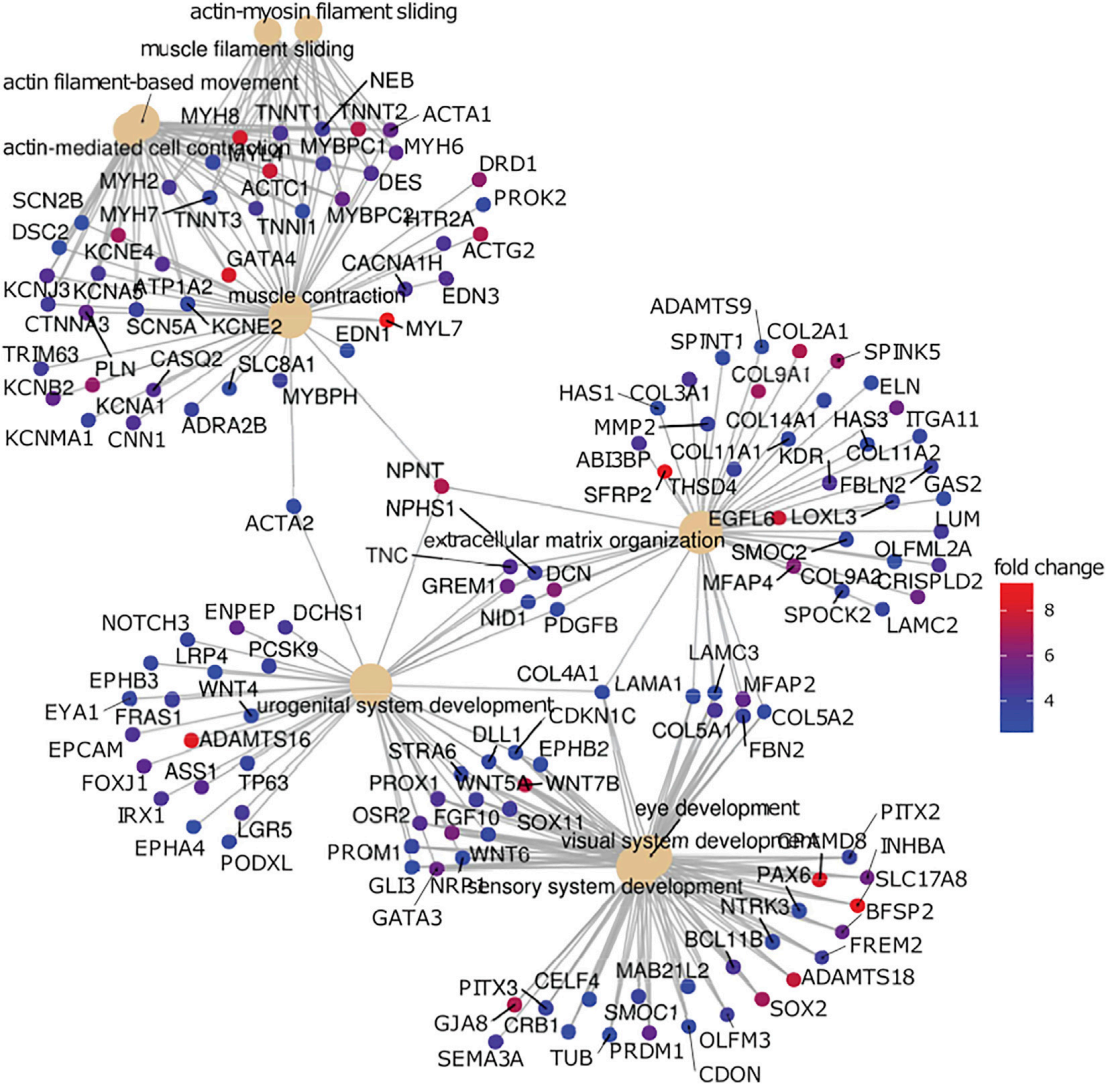
Gene network analysis comparison between RPE. Genes highly expressed in iPSC-RPE when compared to ahRPE are associated with neural differentiation, extracellular matrix organization, and muscle contraction. Genes upregulated in iPSC-RPE versus ahRPE were analyzed for functional roles by gene set enrichment analysis using gene ontology terms (GO). Plot represents the top 15 enriched categories with adjusted *p*-value<0.05 and logFC>2. Categories (GO terms) are plotted as beige nodes, and genes belonging to the respective terms are colored by expression fold change between the groups.

Figure 5 shows a network plot of genes elevated in iPSC-RPE compared to ahRPE at logFC>2 and adjusted *p*-value<0.05, in relation to their top 10 enriched gene ontology categories (GO-BP). The analysis identifies four modules of the highly connected nodes. These were: muscle contraction, extracellular matrix organization, urogenital system development, and visual system development. Some of the most upregulated genes expressed in iPSC-RPE compared to ahRPE in this network were MYL7, SFRP2, ADAMTS16, WNT7B, GATA4, and ACTC1. Altogether, these analyses suggest that cell plasticity is higher in iPSC-RPE, which is consistent with an early eye development expression profile. Considering that the iris muscle also derives from the neuroectoderm of the eye field (Cvekl and Tamm, 2004), the muscle program may not be sufficiently repressed epigenetically in iPSC-RPE, which may partially explain why, when transplanted into patients, iPSC-RPE may contribute to epiretinal membrane formation and proliferative vitreoretinopathy (PVR). Moreover, suppressing these pathways may lead to fostering RPE maturation. If the networks maintaining open chromatin could be suppressed and the contractile apparatus machinery inhibited, RPE identity may be stabilized and plasticity inhibited.

**FIGURE 6:**
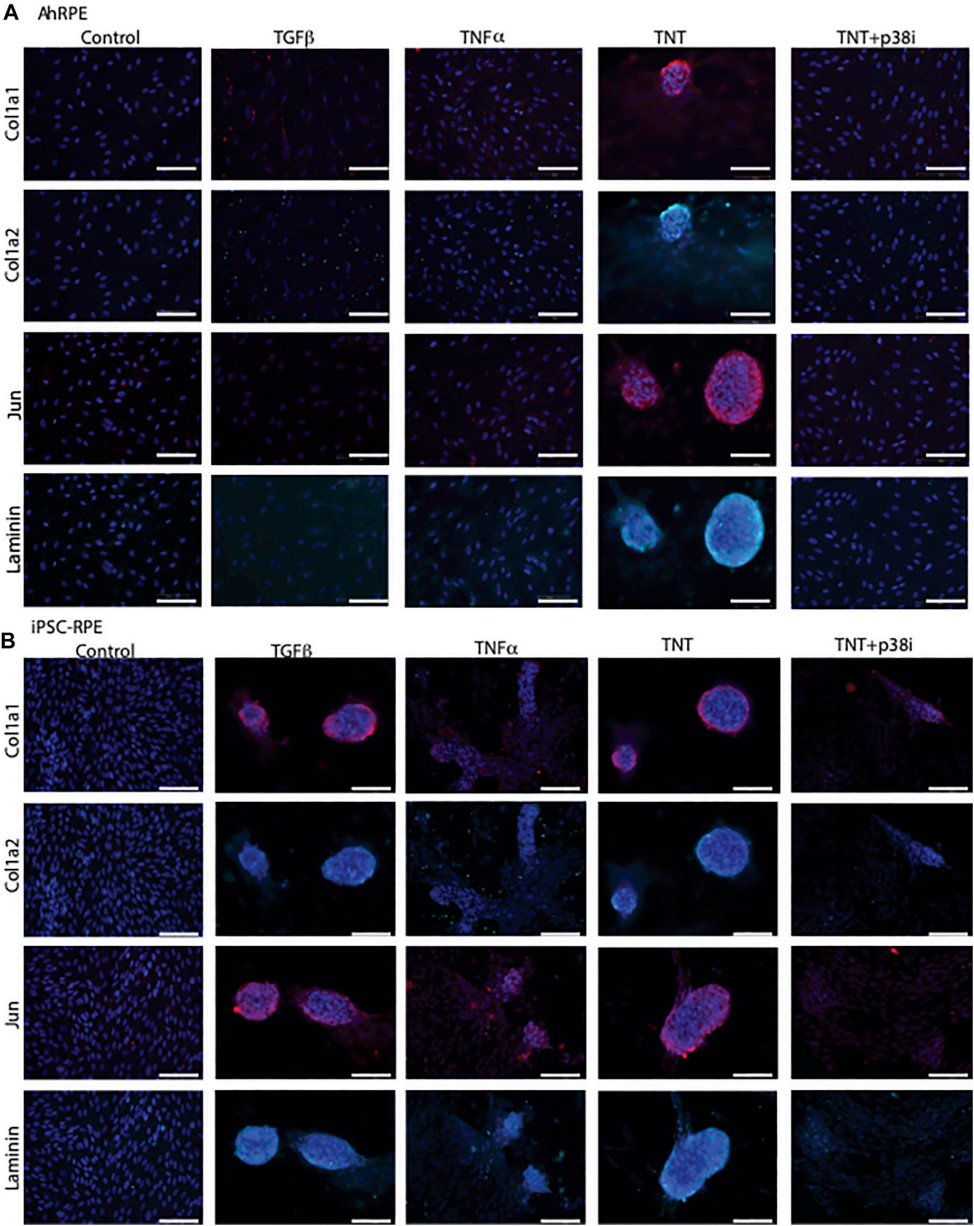
iPSC-RPE contractile aggregates are reduced by p38 inhibition. AhRPE and iPSC-RPE were treated with TGFβ and TNFα, their combination (TNT), or this combination with a p38 inhibitor SB202190 (TNT + p38i). **(A)** AhRPE were fixed and immunostained with the markers of PVR, which included collagen 1a1, 1a2, Jun, and Laminin. **(B)** IPSC-RPE were fixed and immunostained with markers of PVR, which included collagen 1a1, 1a2, Jun, and Laminin. Scale bar = 100 μm.

We previously described an *in vitro* model in which ahRPE are robustly and consistently stimulated to produce contractile membranes (Schiff L, 2019). When ahRPE are treated with TGFβ alone or TNFα alone, they undergo an epithelial to mesenchymal transition. However, when applied in combination, ahRPE are stimulated to produce contractile aggregates (Figure 6A) expressing markers of PVR, which includes Col1a1, Col1a2, Jun, and Laminin. We found that the p38–MAPK pathway was central to the plasticity observed and when inhibited, the activation of the contractile apparatus was prevented, leading to contraction reversal. We sought to compare how iPSC-RPE performed in this same model. We compared ahRPE and iPSC-RPE exposed to TGFβ, TNFα, or their combination. ahRPE will only form contractile membranes in the combination condition. However, iPSC-RPE willingly generate contractile aggregates with TGFβ alone or TNFα alone suggesting an increased sensitivity to transformation (Figures 6A,B). We found that p38 inhibition can inhibit ahRPE from generating contractile membranes (Schiff L, 2019). We examined whether the inhibition of p38 would also inhibit contractile membrane formation in these cells, and we found that contraction membrane formation was inhibited (Figure 6B).

## Discussion

The promise of RPE transplantation for rescuing vision in patients suffering from diseases such as AMD is great, and many scientists are working hard to fulfil this promise. One hurdle in the field is the unintentional transformation in the transplanted cells. When RPE are transplanted in the eye during surgery, many comorbidities known to trigger proliferative vitreoretinopathy are present, which includes a retinal hole, bleeding, inflammation, and cytokine secretion. Here, we conducted a transcriptional comparison between various RPE sources: ahRPE, iPSC-RPE, and ARPE-19 in order to gain insight into the differences in aging, maturation, and plasticity. When comparing iPSC-RPE to ahRPE, we found that the transcriptional differences lay primarily in open chromatin, extracellular matrix remodeling, and early eye development pathways. The data presented here provide clues on how iPSC-RPE may be nudged toward a more differentiated state, but also to prevent RPE from transforming during transplantation.

We previously developed a model to study the mechanisms underlying RPE plasticity and identified that the p38–MAPK pathway was central to RPE transformation (Schiff L, 2019). We tested iPSC-RPE transformation with different combinations of TGFβ and TNFα and found that the iPSC-RPE production of contractile aggregates could be elicited with TGFβ or TNFα alone. This is a significant difference in transformation plasticity compared to ahRPE, which requires the combination of both cytokines for such an aggressive transformation. Nevertheless, like ahRPE, upon p38 pathway inhibition, iPSC-RPE reduced the production of contractile aggregates. Therefore, inhibition of p38 or its partners may aid in stabilizing RPE identity, particularly when exposed to stimuli encountered during the surgical cell transplantation procedure. A more careful study of what transcription factors may be different between the RPE sources is warranted to find additional targets that may improve cell identity stability. The findings gathered by this study may improve the safety of cell therapy not only for RPE but also other epithelia, which undergo fibrotic transformation upon surgical exposure to bleeding and inflammation.

We restricted our initial transcriptional analysis to ahRPE and evaluated whether any individual human donor characteristics was associated with AMD risk or PVR risk SNP alleles, transcriptional expression data, or ahRPE characteristics. We compared these features to each other in addition to TEER, RPE morphology, death to enucleation time, donor age, sex, and morbidity. When ahRPE are cultured in optimal conditions, we could not find any significant differences between any of the risk alleles and any donor characteristics. We did, however, find a negative correlation between death to enucleation time and RPE gene expression signature, and a positive correlation of RPE signature score to TEER (Figures 2C,D). Therefore, the earlier the globes are harvested after donor passing, the higher score the donor receives on the RPE signature index. These results are consistent with the previous reports indicating death to enucleation being critical for maintaining high quality ahRPE (Montanini et al., 2013). Again, our data suggest that TEER is an effective measure of overall RPE fitness (Blenkinsop et al., 2013; Blenkinsop, 2015; Timothy et al., 2015; Fernandes et al., 2018).

The smooth muscle of the pupillary margin is a rare example of muscle derived from the neural tube lineage (Cvekl and Tamm, 2004). PAX6 is the only transcription factor reported to play a direct role in iris muscle development. However, this was also accompanied by other anterior eye phenotypes. Therefore, precisely what PAX6 does to drive iris fate specification and with what partners are still unknown. PAX6 is a highly conserved transcription factor known to specify neural ectoderm and then the eye field, which includes cells of the retina, ciliary marginal zone, the iris epithelium, and iris smooth muscle cells of the pupillary margin (Zhang et al., 2008). Its expression is crucial for proper iris development, as mutations in PAX6 result in iris hypoplasia and delayed iris muscle development (Davis-Silberman et al., 2005; Davis-Silberman and Ashery-Padan, 2008). PAX6 is downregulated in RPE upon differentiation. However, as our data suggest, PAX6 is still elevated in iPSC-RPE compared to ahRPE (Figure 5). A muscle contraction network is also elevated in iPSC-RPE compared to ahRPE. Is the increased expression of these genes maintained by open chromatin not being repressed? Additionally, is the role of ahRPE in proliferative vitreoretinopathy partially due to the shared lineage with iris muscle fate specification? Further studies are needed to answer these questions.

To summarize, we conducted a comparison between 20 primary ahRPE, the immortalized line ARPE-19, and two iPSC-RPE lines. This comparison encompassed morphological scoring, TEER, RPE marker expression, and transcriptional analysis. iPSC-RPE, as reported in the past, possessed the highest TEER, and a similar scoring on RPE signature gene expression. ARPE-19 lagged on TEER and RPE signature gene expression compared to ahRPE and iPSC-RPE. iPSC-RPE expressed genes associated with chromatin regulation at an elevated level compared to ahRPE and ARPE-19 as well as expressing networks involved in early eye development and muscle contraction. Using an *in vitro* model, we demonstrated that iPSC-RPE produces contractile membranes and the production of these membranes can be repressed by p38 inhibition. Future studies will focus on facilitating iPSC-RPE differentiation and suppression of contraction transcriptional programs to facilitate RPE identity stability and cell transplantation safety.

### Limitations to the study

While conclusions can be drawn from the results of the experiments, there are limitations to this study. First, we acknowledge that we have tested only two genetically distinct iPSC lines each differentiated three times (*n* = 6), which have been verified as karyotypically normal. Additional tests using additional genetically distinct iPSC lines would strengthen the power of the conclusions. Including human embryonic cell line–derived RPE and fetal-derived RPE would also be helpful in providing additional context to the maturation state. iPSC variability is a known concern as differentiations from different and even the same lines may result in variable RPE yield. This is why rigorous RPE identity criteria are necessary. However, an additional step that can be taken is to include iPSC-RPE from multiple labs using the same release criteria, which can provide answers to the questions of variability. Considering everything, the results give strong rationale to conduct additional studies on steps that may improve the maturation state of RPE from all the sources to reduce pathological outcomes from transplantation.

## Methods

### AhRPE culture

Human globes from donors aged between 44 and 92 years were obtained from the Eye-Bank for Sight Restoration, Inc, New York, NY, and Miracle in Sights, Winston-Salem, NC. The donors died of lung cancer and myocardial infarction and were negative for all tested serology. A detailed protocol on the isolation and characterization of RPE used for this study has been previously published (Blenkinsop et al., 2013; Fernandes et al., 2018). Briefly, the globes were obtained within 24 h of death. RPE cells were digested with trypsin intraocularly for 50 min. They were then brushed off the Bruch’s membrane, collected, and plated on tissue culture plates coated with 10 μg ml^−1^ Synthemax II (Corning, United States) in RPE medium (Blenkinsop et al., 2013) containing Dulbecco’s modified eagle medium: Nutrient Mix F-12 (DMEM/F12, Gibco), supplemented with 10% heat-inactivated fetal bovine serum (FBS, Sigma), 1X GlutaMAX (Gibco), 1X MEM non-essential amino acids solution (Gibco), 1X penicillin–streptomycin (10,000 U mL^−1^, Gibco), 1X sodium pyruvate (100 mm, Gibco), 10 mm nicotinamide (Sigma-Aldrich), and 0.5X N1 (Sigma-Aldrich), which was changed three times a week.

### ARPE-19

The human retinal pigment epithelial ARPE-19 cell batch used in these experiments was purchased from ATCC, which validates cell authentication to human cell line CRL-2302 (ARPE-19). ARPE-19 were plated on tissue culture plates coated with 10 μg ml^−1^ Synthemax II (Corning, United States) in RPE medium (Blenkinsop et al., 2013) containing Dulbecco’s modified eagle medium: Nutrient Mix F-12 (DMEM/F12, Gibco), supplemented with 10% Heat Inactivated Fetal Bovine Serum (FBS, Sigma), 1X GlutaMAX (Gibco), 1X MEM non-essential amino acids solution (Gibco), 1X penicillin–streptomycin (10,000 U mL^−1^, Gibco), 1X sodium pyruvate (100 mm, Gibco), 10 mm nicotinamide (Sigma-Aldrich), and 0.5X N1 (Sigma-Aldrich), which was changed three times a week. After four weeks, ARPE-19 formed regular cobblestone monolayer.

### Differentiation of hiPSC into RPE

Human induced pluripotent stem cell (hiPSC) lines were grown on irradiated mouse embryonic feeders (Cat. # GSC-6301G; GlobalStem) in a serum-free medium supplemented with 4 ng/ml FGF2 (Cat. # 233-FB; R&D systems). Prior to differentiation, passage 6–26 hiPSCs were feeder-depleted by passaging them onto matrigel-coated plates (Corning) in the presence of mTeSR (Cat. # 05850; StemCell Technologies) medium. The modification of a recently published method (Ferrer et al., 2014) was used to differentiate hiPSC into RPE. Briefly, 100 nm LDN193189 (Cat. # 04-0074; Stemgent) and 10uM SB431542 (Cat. # 1614; Tocris) were added to serum-free differentiation medium every day for three days to drive neural induction from hiPSCs. At day 5 (D5), to specify RPE differentiation, 10 mm nicotinamide (Cat. #N0636; Sigma) and 150 ng/ml Activin A (Cat. # 338-AC; R&D System) were added. Colonies with typical RPE morphology and pigmentation appeared around D30-D40, then the medium was changed to RPE-medium. RPE were then manually picked and plated onto a 24-well plate (Corning, Primaria) at 1-2x105 cells/well. When confluent, the RPE cells were passaged by splitting the monolayer with 0.25% Trypsin-EDTA (Cat. # 25,200–172; Thermo Fisher) supplemented with 12 ng/ml DNase (Cat. # DN-25; Sigma). A pigmented monolayer of RPE was obtained approximately 1 month after splitting, with some variation in time depending on the success of enrichment at each passage. Go-no-go release criteria were used in order to ensure efficient RPE differentiation. A sentinel culture of presumptive RPE was fixed and stained for OTX2 and MITF. Positive staining percentage were calculated. Cultures with >95% positive staining for both OTX2 and MITF was accepted for use in the study. RPE differentiations failing this criterion were either not included or repurified using steps outlined by Khristov et al., (2018). Validated hiPSC-RPE were replated onto transwell inserts (Corning) for 12-well plates or onto 24-well plates (Cat. # 3527; Corning) at 130,000 cells/well or onto 48-well cell culture plates (Cat. # 3548; Corning) at 50,000 cells/well for further analysis. Statistical analysis was conducted using the Student’s t test.

### Genotyping

TaqMan genotyping assays (Thermo Fisher) were used for genotyping SNP rs10490924 in the *ARMS2* gene (Assay ID: C__29934973_20; Cat. # 4351379), SNP rs1061170 in the *CFH* gene (custom SNP genotyping assay; Assay ID: AHI1TPW; Cat. # 4331349), SNP rs2230199 in the *C3* gene (Assay ID: C__26330755_10; Cat. # 4351379), SNP rs243845 in the MMP2 gene (Assay ID: C___3225954_10; Cat. #), SNP rs7226755 in the SMAD7 gene (Assay ID: C__29019553_10; Cat. #), rs1800471 in the gene TGFB1 (Assay ID: C__11464118_30; Cat. #), rs429358 in the gene APOE (Assay ID: C___3084793_20; Cat. #), rs7412 in the APOE gene (Assay ID: C____904973_10; Cat.#), and rs2229094 in the TNF gene (Assay ID: C___2451908_10; Cat.#). Statistical analysis was conducted using the Student’s t test.

### Transepithelial electrical resistance

RPE cells from ahRPE, ARPE-19, and iPSC-RPE cultures were replated onto Synthemax II (10 ug/ml, Cat. # 3535; Corning) coated transwell inserts (Cat. # 3460; Corning) at a density of 1 × 10^5^ cells per well. RPE media of the same formulation described in ahRPE methods section was changed three times/week, for four weeks. TEER was measured using the EVOM2 epithelial volt–ohmmeter (World precision instruments). Statistical analysis was conducted using the Student’s t test.

### Immunocytochemistry for RPE markers

RPE were fixed on transwell inserts (Cat. # 3460; Corning) and in 24- or 48-well cell culture plates (Cat. # 3527 and Cat. # 3548; Corning) using 4% paraformaldehyde (Cat. # sc-281692; Santa Cruz) in PBS for 10 min, followed by rinsing three times with PBS. The fixed cells were blocked and permeabilized for an hour in a solution consisting of 0.01% Saponin (Cat. #S7900; Sigma) and 5% normal goat serum (Cat. # 005-000-121; Jackson ImmunoResearch) in 1% BSA (Cat. # sc-2323; Santa Cruz). The fixed cells were then incubated overnight at 4_C with primary antibodies for OTX2 (Cat. # Ab92515; Abcam) and ZO-1 (Cat. # 716,300; Invitrogen). The cells were rinsed three times with PBS and incubated with the secondary antibodies [OTX2 and MCT1: Goat anti-Rabbit IgG (H + L) Secondary Antibody: Cat. # A11035 and A21245; Thermo Fisher].

### RNA isolation and Quality Control

Human RPE cells were lysed in 350uL of RLT+ buffer containing 1% β-Mercaptoethanol. Total RNA was extracted using Qiagen AllPrep DNA/RNA/miRNA Universal Kit (80224). Total RNA samples were quantitatively and qualitatively assessed using the fluorescence-based Broad Range Quant-iT RNA Assay Kit (Thermo Fisher Scientific) and the Standard Sensitivity RNA Analysis DNF-471 Kit on a 96-channel Fragment Analyzer (Agilent), respectively. Concentrations averaged at 30 ng/μL while RIN ranged from 8.6 to 10.0, with a median at 9.7.

### mRNA sequencing

32 human RPE-derived RNA samples were normalized on the MicroLab STAR automated liquid platform (Hamilton). Total RNA input of 200ng was used for library construction with the NEBNext Ultra II Directional RNA Library Prep Kit for Illumina #E7760, together with the NEBNext Poly(A) mRNA Magnetic Isolation Module #E7490 upstream and the NEBNext Multiplex Oligos for Illumina #E7600 downstream (all New England Biolabs). The only deviation from the manufacturer's protocol was the use of Ampure XP beads (Beckman Coulter) for double-stranded cDNA purification, instead of the recommended SPRIselect Beads. The index PCR was performed with 12 cycles, while the final library was eluted in 30μL. mRNA libraries were then quantified by the High Sensitivity dsDNA Quanti-iT Assay Kit (ThermoFisher) on a Synergy HTX (BioTek). Library molarity averaged at 34.7 nM. mRNA libraries were also assessed for size distribution (smear analysis of 322 bp average) and adapter dimer presence (<1%) by the High Sensitivity Small Fragment DNF-477-33 Kit on a 48-channel Fragment Analyzer (Agilent). All 41 sequencing libraries were then normalized on the MicroLab STAR (Hamilton), pooled and spiked in with PhiX Control v3 (Illumina). The library pool was subsequently clustered on a HiSeq 3000/4000 SR Cluster kit and sequenced on a HiSeq 4000 Sequencing System (Illumina) with dual index, single 85 bp read (Read parameters: Rd1: 86, Rd2: 8, Rd3: 8), reaching an average depth of 31.5 million Pass-Filter reads per sample (8.4% CV).

### Data processing and analysis

Raw data from the RNA-seq experiment were processed with a pipeline based on the ENCODE “Long RNA-seq” pipeline. Filtered reads were mapped against the *Homo sapiens* (human) genome hg38/GRCh38 (primary assembly, excluding alternate contigs) using the STAR aligner software (STAR version 2.5.2b) [33] allowing for soft clipping of adapter sequences. The quantification of transcript levels was performed using RSEM (RSEM version 1.3.0) [34] and featureCount (featureCount version 1.5.1) [35], with annotation from Ensembl95. Quality controls were implemented using FastQC (FastQC version 0.11.5) [36], picardmetrics (picardmetrics version 0.2.4), and dupRadar (dupRadar version 1.0.0) [37] at the respective steps. Normalized log2 Counts Per Million (CPM) mRNA expression values were calculated *via* the voom function provided by the limma R package (limma version 3.42.0).

Initial quality control found three samples with mismatched sex-specific gene expression, and these were removed from the analysis. Principal component analysis was performed using the prcomp routine, on all samples as well as on the ahRPE samples only. In order to detect potential confounding factors within the ahRPE samples, we used ANOVA tests comparing a list of potential confounders such as donor age, sex, time to enucleation, sample preparation date, and RIN against variables including the first five principal components as well as phenotypic read outs (TEER score and morphology grade). To test the influence of the SNP status on the expression within the primary ahRPE samples, linear regression models were built for each individual SNP status (NN versus NR, NN versus RR, and NN versus not-NN) as well as for clustering of the genomic data into two groups (clustering generated by kmeans). Additionally, we used the MatrixEQTL package to test for significant associations between SNP status and expression (including covariances donor age, sex, time to enucleation, and time to preservation). Due to the variability of the genomic status of the primary cells and the resulting low power, we did not detect significant and stable associations. We then sought to measure the molecular identity of the samples using an RPE tissue expression signature published in Strunnikova et al. (2010). We measured the expression of the 154-gene signature on the samples using the ssgsea method in the gsva package. We calculated the correlation of the resulting scores with the principal components and plotted the colors onto the PCA. For validation, we computed the ssgsea scores for >4,000 gene signatures from GO, computed the correlation between these and the principal components, and ranked the RPE signature within this list. We then calculated and plotted ssgsea values for the RPE signature for all samples by group (Kruskal–Wallis test). To test whether the RPE signature scores indeed aligned with morphology grade, TEER scores, and other features, we used linear regression and ANOVA test.

In order to assess the difference between the source types, we selected ahRPE samples that satisfied donor age <75 years, RPE morphology grade >3.5, and RPE-154 score >0.6. These samples were considered phenotypically and molecularly representative of normal human RPE tissue. Within these, we computed differentially expressed genes using limma-voom, with a cutoff of FDR<0.05 and logFC>0.5. Overlap between the gene sets was tested for significance and plotted using the super exact test package (SuperExactTest v1.0.7). For further analysis of these genes, we selected well-expressed genes (mean TPM >10) and created lists of up- and downregulated genes for each source type comparison as input for the R package clusterProfiler. The short lists were created using stricter cutoffs of abs (logFC)≥1. Because of the uneven number of DEGs between the contrasts we used a logFC cutoff of 2 for the IPSC-versus-ahRPE upregulated gene list. We computed enrichments for the gene lists using the clusterProfiler enrichment functions. Gene sets were from MSigDB v7.1 and included GO terms (c5) and KEGG/BioCarta/PID/REACTOME (c2.cp). Enriched categories were plotted using the package routines. The compareCluster method was used to create dotplots of the enriched categories across the source type comparisons. Gene signature expression heatmaps were created using GSVA (method gsva) with the gene sets (GO) followed by t-welch test (*t*-test with unequal variance, row_t_welch) with *p*-value adjustment (BH), ranking, and filtering (mean difference >0.5). Heatmaps were plotted using the pheatmap package. All analyses were performed in R v3.6.1.

## Data Availability

Sequencing data has been submitted to the GEO database and is available under the accession number GSE210331.
