# Supplementary material for: Transcriptional comparison of adult human primary Retinal Pigment Epithelium, human pluripotent stem cell-derived Retinal Pigment Epithelium, and ARPE19 cells
Source: Front Cell Dev Biol. 2022 Aug 26;10:910040. doi: 10.3389/fcell.2022.910040 (PMC9461284; doi:10.3389/fcell.2022.910040)
Supplement: Supplementary file 1 [file DataSheet1.docx]

Supplemental Material

Supplemental Figure 1

Supplemental Figure 1. RNA sequencing transcript comparison. A) Comparison of RNA integrity between RPE sources. B) Total number of genes expressed based on RPE sources.

Supplemental Figure 2


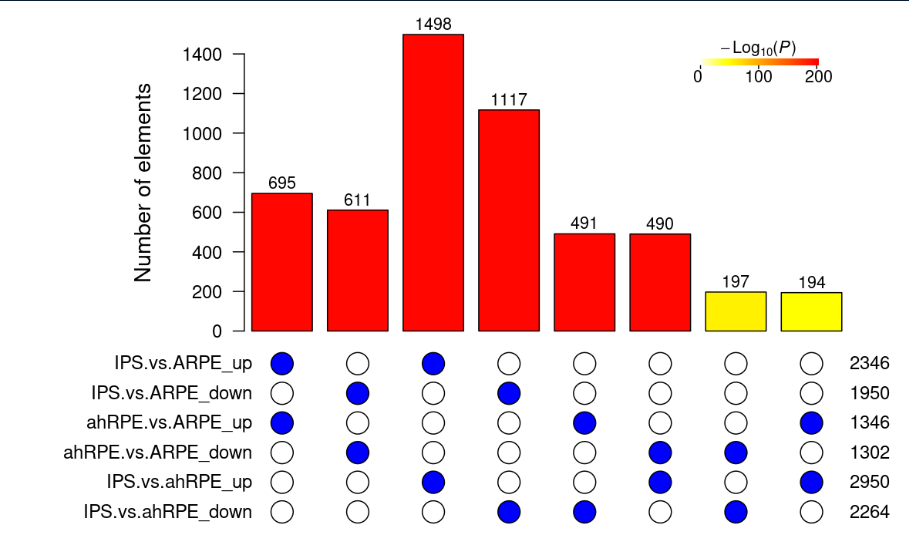


Supplemental Figure 2. Overlap between differentially expressed gene sets in the different comparisons. Plotted are the numbers of genes in overlaps between the pairs of comparisons shown in blue. Total numbers of DE genes in each comparisons are listed on the right of each comparison name, number of genes in overlaps are plotted and exact values indicated above boxes, significance of the overlap (superexact test) is indicated in the color scheme.
